# Par6 family proteins in cancer

**DOI:** 10.18632/oncoscience.255

**Published:** 2015-10-07

**Authors:** Elsa Marques, Juha Klefström

**Affiliations:** Research Programs Unit/Translational Cancer Biology, University of Helsinki, Helsinki, Finland

**Keywords:** tumorigenesis, Par6 proteins, proliferation, polarity

The regulatory networks of cell polarization and polarity effector proteins have been subjects of feverish interest in the field of cell and developmental biology but many advances in the polarity research may have flown unnoticed past the radar of mainstream cancer biologist. However, recent findings suggesting important cell cycle gatekeeping functions for polarity proteins may change that. For example, PAR6 proteins interact with classical cancer driver signaling pathways, including MAPK and PI3K and moreover, PAR6 (PARD6) genes are frequently altered in various cancers. Mammalian genomes harbor three different PARD6 genes. Recent studies in breast cancer have suggested that different PARD6 genes are not only important players but may play even opposite roles during tumorigenesis.

Par-6 is one of the partitioning-defective (par) genes identified by Kemphues et al in a landmark genetic screen, which discovered genes important for the first, asymmetric embryonic cell division in C.elegans [1]. In the invertebrate oocytes, most Par proteins are segregated to two opposite poles of the cells - the anterior cell cortex is occupied by Par-3 and Par-6, which interact with each other via PSD95/Dlg/ZO1 (PDZ) domains. In epithelial cells of diverse species, including humans, the PAR3/PAR6 defines the apical region and co-localizes with tight junctions. A current view is that PAR6 is a multimodular scaffold protein, which together with PAR3 forms a loose or non-constitutive complex with atypical protein kinase C (aPKC) and CDC42. This PAR3-PAR6-aPKC/CDC42 or ‘PAR complex’ has a principal role in most if not all process where cellular asymmetry is important, for example asymmetric cell division, apico-basal and anterio-posterior polarity, axon specification and directional migration [2, 3].

The evolution's kitchen has added an extra degree of complexity to the polarizing systems in large and long-lived animals by multiplying the single Par-6 gene in invertebrates to three separate PARD6 genes in mammals: PARD6A, PARD6B and PARD6G. In humans and mice, all PARD6 genes reside in different chromosomes yet they are very similar, with > 70% sequence similarity. Nevertheless, many PAR6 studies do not clearly indicate which of the three proteins was studied and not surprisingly, the picture of individualistic functions of PAR6 proteins has remained incomplete.

Recent findings in breast cancer research have suggested intriguing differences between the PARD6 genes. PARD6B locus resides in a chromosomal region that is frequently amplified and overexpressed in breast cancer [4]. In cell culture, PAR6A-aPKC activity is important for HER2/ErbB2-dependent disruption of organized epithelial structure [5]. Moreover, elevated PARD6A or PARD6B expression signals together with aPKC (PKCɩ) and CDC42 to stimulate MAPK signaling and cell proliferation, without affecting the apico-basal polarity [4]. Thus, the genetic and functional evidence suggest a gain-of function i.e. oncogenic mode of activity for PARD6B in breast cancer.

Contrary to the earlier findings, our shRNA screen identified both PAR6B and PAR6G as critical suppressors of cell proliferation [6]. The study was designed to identify genes important for epithelial cell cycle restriction in three-dimensional mammary epithelial organoid culture. MCF10A cells undergo morphogenesis to form quiescent acinar structures in basement membrane gels. The neighborhood suppression of proliferation is tight in matured structures, able to resist even the cell autonomous proliferation signals from oncogenic Myc. The tumor suppressor gene LKB1 has been earlier identified as a key proliferation gatekeeper in mature MCF10A organoids [7]. LKB1 is a human homolog of par-4 (one of the Kemphues genes) and therefore, it was thrilling to discover similar cell cycle gatekeeping functions in other PARD genes [6]. Loss of PAR6G could alone prevent the epithelial cell cycle restriction, whereas loss of PAR6B needed cooperation with oncogenic Myc to trigger the cell cycle re-entry in quiescent MCF10A structures. Loss of PAR6B or PAR6G diminished the phosphorylation of PKCz, which indicates decreased PAR complex activity and the gene deficiencies enabled growth factor-independent cell cycle progression in monolayer culture. The PAR6B or PAR6G-deficiency induced cell cycle deregulation associated with strong AKT phosphorylation on T308, which residue is used for AKT phosphoactivation by PI3K/PDK1 pathway. Therefore, PAR6 activity appears to be important for repressive regulation of PI3K/PDK1/AKT-dependent proliferation signaling [6].

**Figure 1:**
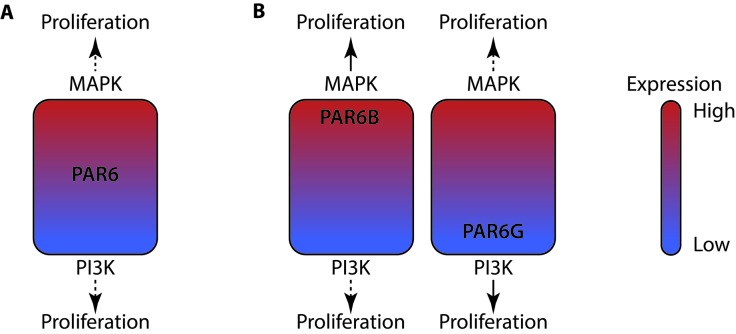
PAR6 proteins play opposite roles in tumorigenesis **A.** In mammary epithelial organoid culture, augmented PAR6 expression promotes MAPK activity whereas diminished expression induces PI3K/AKT pathway. This duality could explain why PAR6 up- and downregulation both result in a similar output: promotion of cell proliferation and corruption of the neighborhood suppression of proliferation. **B.** However, the tumorigenic processes strongly favor PAR6B upregulation and PAR6G downregulation, which difference is more likely related to mechanisms of tumorigenesis than to biological capacity of different PAR6 proteins.

Should we interpret these findings so that any shift in PAR6 activity, up or down, predisposes to uncontrolled cell proliferation and tumorigenesis? The question was addressed by investigating cancer genomes for the type of mutations affecting different PARD6 genes. Consistent with the suggested oncogenic role of PAR6B, chromosomal gains, amplifications and overexpression dominated the landscape of PARD6B mutations. The surprising finding was that PARD6G landscape was etched with typical loss of function mutations: chromosomal losses, deletions and loss of heterozygosity. Thus, PARD6B and PARD6G genes display essentially opposite mutational landscape in tumors. We posit that while a change in any individual PAR6 protein has the capacity to bring PAR6 activity up- or downwards in the cells to instigate specific cell proliferation pathways, for some reason the tumorigenic processes seem to favor gains for PARD6B and losses for PARD6G (Fig.1). The differences could arise from cell type-specific expression, subcellular compartmentalization of the protein or even from different context-dependent functions of PAR6 proteins in tissues, which will eventually determine in which way an expression-altered PAR6 protein best serves the process of tumorigenesis. Future studies will be needed to better clarify the roles of individual PAR6 genes in tumor development – which surely will be an exciting research area offering perspectives to evolutionary biology and oncology alike.
